# On the Anemia of Pregnant Females

**Published:** 1853-01

**Authors:** 


*On the Anemia of Pregnant Females.* By Geo. Martin, M. D., of Delaware County, Penn. (Read before the Delaware County Medical Society.)

Every obstetrical writer mentions plethora connected with pregnancy as the source of some of those diseases, which, when they occur, so often compromise the safety of both mother and child.

Much has been said about it, and so little about anemia, that it has been and is yet considered by some practitioners as the almost constant complication of pregnancy. The bare announcement of a poor woman being pregnant, has been to them clear evidence that she was plethoric, and a sufficient warrant for the use of the lancet; and to so great an extent has this been carried, that many females, even at the present day, think that they cannot be delivered of a healthy child, and themselves do well, if they have not been bled once or twice before the pains of parturition come on. Moreover, the physician who has been called upon to perform the operation, if he should chance to think differently, and have the hardihood to stand by his opinion, will find, in the event of a misfortune occurring either to the mother or child, that the blame will rest upon his shoulders; and there will be persons, and those not a few, who will not hesitate to tell him that the result would have been otherwise if he had not refused to bleed.

Now this is certainly wrong; for though no one will deny that plethora does exist in some instances, yet it does so much more rarely than anemia; and I shall here endeavor to show that most of these cases in which bleeding is resorted to, arise from an impoverished blood, and that the use of tonics and even of the chalybeates would be followed by the happiest results.

Are the nausea, vomiting, and depraved appetite that are so common, and occupy so large a portion of gestation, symptoms to lead to suspicion of danger from an over supply of nutriment? On the contrary, if such derangements of the digestive apparatus were met with at any other time, would we not immediately try to relieve them, for fear that debility, emaciation, and even death might ensue? All medical experience points to such a course; and why should not the result be the same in one case as well as the other. There surely is nothing mysterious hanging over the pregnant female that will reverse all our known laws of the animal economy.

It has been said that the stoppage of the menstrual secretion counteracts these influences, and produces the supposed plethora; but this can hardly be, for the amount of nutriment drawn from the blood in forming a highly-organized living being weighing from seven to eight pounds, with two or three more of appendages, must far exceed the amount required for nine catamenial periods.

The symptoms calling for depletion, as they are commonly described, are headache, vertigo, flushes of heat, depression of spirits, a full, frequent pulse, a feeling of fullness and pain in the pelvic region, and a tendency to hæmorrhages in various parts of the body. Now it will be easy to show that many of these are often produced by anæmia; and though bleeding may relieve some of them for a time, they will be sure speedily to return, as the remedy only aggravated that condition of the circulating fluid in which they originated; and to this may be attributed that necessity of a frequent resort to the lancet which many practitioners will tell us they have found. Anæmia also has a strong tendency to derange the circulation; for when the blood is in this condition, it does not carry with it that stimulus which is necessary to excite the capillaries to do their part, and congestion will frequently ensue.

The nervous excitability, too, is greatly increased by this condition of the system, and this is the common cause of simple neuralgia, which it may produce in two ways: first, by its not being stimulating enough to the nervous centers to maintain a healthy action in them; and secondly, by its not affording the different organs a proper amount of good plasma to keep them in a healthy condition, and they demand through the nervous system a better supply. This also is the reason of that frequent, easily excitable, and sometimes full pulse which is so often met with in pregnancy and anæmia; for the heart here, in endeavoring to answer the demands made upon it, acts much more rapidly than it does in health, and as these wants are augmented in proportion to the amount of labor that is to be performed, we shall find that the least exertion will be followed by a great increase in the number of pulsations. During gestation, this excitability is manifested to a great degree, though in this case it is not to be attributed solely to the impoverished blood, for the nervous disturbances created by the changes wrought in the womb after conception tend considerably to increase it.

If we investigate the manner in which the above symptoms are produced, we shall find when they may be considered as evidences of plethora and when not. To begin we will take the pain in the head, which, when not sympathetic with some other disorder, often arises from neuralgia, sometimes from congestion at others from irritation, and occasionally from inflammation. Now, I have before stated that anæmia is the common cause of neuralgia, and I have shown that it strongly predisposes to congestion, which will be liable to take place whenever there is any excitement or irritation of the brain; and this may occur in any condition of the system. Yet, the result will be very different in the two cases; for when plethora exists, we shall have inflammation immediately following the congestion; whereas, in anæmia, it it may exist for some time without it. And this is very true in pregnancy; for here we sometimes have it lasting for weeks; producing much suffering, and at times followed by the most disastrous results, without our being able in many cases to discover a symptom of inflammation during life, or a trace of it after death; and when it does occur, it will generally be found to have arisen from some direct injury which the overloaded vessels have inflicted upon the tissue of the organ. Vertigo generally arises from it, as does also the disposition to hæmorrhages, when they do not occur from the vitiated blood relaxing the vessels, and thereby obtaining a free exit; and the sense of pain and fullness in the pelvic region must be attributed to the same cause affecting the womb.

The flushes of heat and other nervous symptoms not unfrequently met with proceed from the excitability before treated of as arising from a deficient plasma; and as this must necessarily have a great influence over the moral junctions, too, it will account for that depression of spirits so common in gestation.

From the foregoing facts, it will require no force to arrive at the conclusion that the symptoms of anæmia greatly preponderate in the above; and to them I shall add a few others which will serve to place the subject in a still clearer light. Thus, the face often appears somewhat bloated and discolored; the blood, when drawn, frequently presents the buffy coat without their being the least evidence of inflammation existing at the time; the carotids throb, and at times even a partial loss of vision ensues. Now, these are all known as symptoms of anæmia; and the latter has been frequently produced by an extensive hæmorrhage. In an analysis of the blood by Andral and Gavarret (for the account of which I am indebted to Caseaux's work on Midwifery,) thirty-two cases out of thirty-four were found in which the globules were below the healthy mean, in six of which they ranged from 120 to 125; and in twenty-six from ninety-five to 120; they also found for the first six months the fibrine in the thirty-four cases was uniformly below the natural quantity, varying from 1-9 to 2-9; while during the last three months it exceeded it, ranging from 2-9 to 4-8, and averaging nearly 4; this, therefore, gives the true reason for the buffy coat; and we here have thirty-two cases out of thirty-four in which anæmia was proved to exist.

The treatment of this affection should, of course, consist of tonics, the best of which are the ferruginous preparations, a nutritious diet, fresh air, and moderate exercise; the nervous symptoms, when excessive, should be allayed by the antispasmodics and opiates; and when congestion occurs, it will generally be speedily relieved by cold applications to the part, counter-irritants and rest, with a little aperient medicine if the bowels should be constipated; and perhaps it may not be out of place to mention here, that when the brain is the seat of engorgement, the best mode of applying the cold to it will be by pouring water of a low temperature in a steady stream upon the head, care being taken to watch the patient, that she be not too much prostrated thereby. It would be easy to cite numerous instances in which the above treatment has relieved headache, vertigo, and even convulsions, in some of which after depletion had been fairly tried and failed; but I trust enough has been said to show the real character of the complaint, and with a few words upon the injuries that a false diagnosis may inflict upon the child, I shall bring this subject to a close.

It is an acknowledged fact that tubercles and scrofula often originate in a depraved condition of the system, and as the vital organs of the fœtus at the time of their formation are undoubtedly in their most delicate condition, and very susceptible of change, may not the imperfect plasma, which is all the parent can furnish out of her impoverished blood, lay the foundation for these diseases, which only wait for some exciting cause to call them into action? And if this be the case, how highly important it is, both to the mother and her offspring, that the true nature of the affection be clearly understood, and the proper remedies at once applied.—*Am. Jour. of Med. Sciences.*

---

Dr. Condie inquired of the Fellows present, whether they had any experience in reference to the effects of quinia in large doses in the treatment of typhoid fever. He had himself employed it in five cases occurring in children, in all of which the characteristics of the disease were distinctly marked. The fever was in no instance cut short by the quinia; but it certainly appeared to him, that under its use, the duration of the disease was very materially shortened, and convalescence more promptly and fully established. The quinia was given in solution, in doses of from two to three or four grains, every three hours, according to the age of the patient, combined with one-third the quantity of tannic acid, by which the intense bitterness of the quinia was in a great measure removed, and one great difficulty in the administration of the remedy to young patients done away with.—*Quarterly Summary of Trans. of College of Physicians of Philadelphia.*

